# HPV16 E6 seropositivity and oropharyngeal cancer: Marker of exposure, risk, or disease?

**DOI:** 10.1016/j.ebiom.2020.103190

**Published:** 2021-01-06

**Authors:** Rebecca Landy, Anil K. Chaturvedi

**Affiliations:** Clinical Epidemiology Unit, Clinical Genetics Branch, Division of Cancer Epidemiology and Genetics, National Cancer Institute, National Institutes of Health, 9609 Medical Center Drive, Rm. 6-E238, Rockville, MD 20850, United States

Human papillomavirus (HPV) infection, primarily HPV16, is responsible for a substantial increase in the incidence of oropharyngeal cancers (base of tongue, tonsil, soft palate, and pharyngeal wall) among men in recent years in several developed countries [1]. Recent studies have discovered HPV16 E6 seropositivity (IgG antibodies measured through a Luminex-multiplexed assay) as a biomarker strongly associated with prospective oropharyngeal cancer risk [2,3]. Yet, little is known regarding HPV16 E6 seroepidemiology in the general population.

In the December issue of *EBioMedicine*, Brenner et al. [4] report on the first large population-based study of HPV16 E6 seroepidemiology within the UK Biobank (ages 40–69 years). The key results include: 1) low prevalence of HPV16 E6 seropositivity (~0.8%); 2) seropositivity was associated with several markers of high-risk sexual behaviours; and 3) seropositivity was not associated with age, gender, or smoking status.

Herein, we place the results of Brenner et al. [4] within the context of the field with respect to the biological/epidemiological meaning of HPV16 E6 seropositivity and the clinical implications for screening.

*Is E6 seropositivity a marker of exposure, risk, or disease?* Brenner et al. [4] report higher E6 seropositivity in individuals with a higher number of lifetime sex partners, same-gender sex partners, early sexual debut, and in those seropositive for other sexually transmitted infections. These associations imply E6 seropositivity is a marker of exposure, consistent with acquisition of HPV infections at multiple anatomic sites, primarily through sexual activity [5].

Serologic markers of HPV, such as L1 antibodies, generally indicate cumulative exposure invariant of the site (i.e., anogenital vs. oral) or duration of infection (i.e., short-term vs. long-term) [5]. By contrast, accumulating evidence suggests E6 antibodies are specific for oral/oropharyngeal HPV infections. For example, HPV16 E6 seropositivity is specifically associated with risk of oropharyngeal cancer, and to a limited extent anal cancer, but not cervical, vaginal, vulvar, or penile cancers [2,3,6]. Such anatomic specificity potentially reflects the discontinuous basement membrane in the tonsillar/base of tongue crypt epithelium, the site of origin of HPV-positive oropharyngeal cancers, which enables immune recognition of HPV [7].

HPV16 E6 antibodies also appear to mark persistence of oral/oropharyngeal HPV infections, given that population-level E6 seropositivity is low relative to oral HPV16 incidence (~1% annual incidence) [8]. The temporality of E6 seroconversion in the natural history from oral HPV infection to the development of HPV-positive oropharyngeal cancer is, however, unclear. Two observations support E6 seroconversion as an early event, closer to the establishment of oral HPV infection. First, the observation in prior studies that E6 seroconversion precedes cancer diagnosis by several decades [2,3]. Second, the similarity in E6 seroprevalence by age (40–69 years) reported by Brenner et al. [4]. Presumably, most oral HPV infections that lead to E6 conversion are acquired prior to age 40, and thus E6 seroprevalence remains stable at older ages.

Alternatively, recent data suggest E6 seroconversion represents an increasingly late event in the natural history continuum. For example, Kreimer et al. [2] reported that among incident oropharyngeal cancers, E6 seropositivity increased with decreasing sampling-latency (time between blood sampling and cancer diagnosis), reaching a peak ≤ 5 years prior to cancer diagnosis. However, under such a natural history construct, population-level E6 seroprevalence would be expected to increase with age and peak around the ages of peak oropharyngeal cancer incidence. Further, E6 seroconversion closer to cancer diagnosis would imply variable relative risks (RR), positive predictive values (PPV, the probability of disease in seropositive individuals), and sensitivity for E6 (vs. HPV-positive oropharyngeal cancer) across disease-latency intervals.

HPV16 E6 seropositivity is associated with >100-fold increased risk of oropharyngeal cancer, making it a unique biomarker [2,3]. Yet, the low PPV (~0.6%−0.7% per year) [3] argues against E6 seropositivity as a biomarker of underlying disease. Indeed, E6 seroprevalence in a population (1 in 125 individuals) far exceeds annual oropharyngeal cancer incidence (1 in ~10,000 individuals).

Collectively, the observations of Brenner et al. [4] and the existing literature suggest that HPV16 E6 seropositivity is low in the general population, indicates oral/oropharyngeal HPV exposures (and that such exposures occur prior to age 40 years), marks high relative risk but low absolute risk of future oropharyngeal cancer, and is unlikely to represent a biomarker of prevalent disease. However, a few unexplained observations by Brenner et al. [4] indicate epidemiologic incoherence between HPV16 E6 seroepidemiology and oropharyngeal cancer epidemiology—the lack of association of E6 seropositivity with male gender and smoking, two strong, independent risk factors for oropharyngeal cancer (both HPV-positive and negative) [1]. Further compounding such incoherence, oral HPV prevalence and persistence are higher in men versus women [9,10]. As an explanation for similar E6 seroprevalence yet dissimilar oropharyngeal cancer incidence in men and women, Brenner et al. [4] suggest that the anatomic source of E6 seropositivity could be differential by gender (oral infections in men and anal infections in women).

Instead, we propose that a similar E6 seroprevalence by gender (or smoking) could arise from similar early natural histories (e.g., oral HPV16 incidence and short-term persistence) and/or dissimilar immune responses relevant for E6 seroconversion (e.g., more robust in women vs. men, akin to L1 antibodies [5]). In turn, a similar seroprevalence and relative risk (RR) for E6 seropositivity—oropharyngeal cancer in both genders translates to a dissimilar PPV (higher in men) because of differential background rates of oropharyngeal cancer incidence in E6 seronegative men and women (complement of the negative predictive value [cNPV]) [3].

*Clinical implications for screening*: The promise of HPV16 E6 seropositivity notwithstanding, the lack of an identifiable HPV-induced precancer in the oropharynx means screening for secondary prevention is not currently possible [1,5]. Likewise, early detection remains questionable because the benefits and harms of screening remain unknown. Thus, E6 serology remains a marker to rule-out HPV-positive oropharyngeal cancer. Additional research on screening modalities and biomarkers is needed to rule-in disease in E6 seropositive individuals. Nonetheless, E6 serology represents a promising biomarker to enrich clinical studies on screening methods and risk-mitigation strategies.

In conclusion, the study by Brenner et al. [4] represents an important advance in characterizing the population-level epidemiology of HPV16 E6 seropositivity. Long-term natural history studies with multiple time-point biospecimen sampling for E6 serology and multiple-anatomic-site HPV infection (DNA-based) are needed to elucidate the temporality of E6 seroconversion in multistep HPV-positive oropharyngeal carcinogenesis. Admittedly, such studies would require tens of thousands of participants, given the rarity of HPV16 E6 seropositivity in the general population.

## Funding sources

Intramural Research Program, 10.13039/100000054National Cancer Institute, 10.13039/100000009National Institutes of Health.

## Author contributions

**Conception**: Landy and Chaturvedi

**Drafting, critical revision, and final approval of the manuscript**: Landy and Chaturvedi.

## Declarations of Competing Interests

The authors do not have any relevant conflicts of interest to report.
